# Estrogens drive myeloid-derived suppressor cell accumulation

**DOI:** 10.18632/oncoscience.340

**Published:** 2017-02-24

**Authors:** Jose R. Conejo-Garcia, Kyle K. Payne, Nikolaos Svoronos

**Affiliations:** Department of Immunology, H. Lee Moffitt Cancer Center and Research Institute, Tampa, FL 33612, USA

Estrogen, the primary female sex hormone, drives the progression of 70-80% of breast cancers and has a poorly investigated role in other malignancies. Estrogens' transcriptional effects are mediated by two high-affinity nuclear receptors: ERα and ERβ, although ERα is considered the dominant isoform and only its expression is usually assessed.

Following multiple worldwide trials in the 1980's, the selective ER modulator (SERM) tamoxifen has been effectively used to reduce both recurrence and mortality in patients with ER+ breast tumors. Importantly, tamoxifen can have estrogen agonistic effects in other cell types, including the endometrium [1]. To prevent these paradoxical activities during the treatment of cancer, alternative interventions have been implemented. Fulvestrant, for instance, is a selective ER degrader (SERD) with no agonist effects. In addition, aromatase inhibitors are used in postmenopausal women because estrone, produced by androgen conversion by the aromatase hormone, is the predominant circulating estrogen after menopause.

Besides tumor cells and cells from the reproductive tract, hematopoietic progenitors, including precursors of lymphoid and myeloid cells, also express both ER isoforms. To understand how estrogen signaling influences both tumor-promoting and protective anti-tumor immune responses, independent of direct signaling on tumor cells, we tested the effects of oophorectomy and/or *ad libitum* estradiol supplementation using a variety of estrogeninsensitive transplantable tumor cells [2]. Our results demonstrate that estrogen signaling has a slight inhibitory activity on tumor-reactive T cells. However, those effects cannot explain the significant differences in malignant progression observed in our systems. In contrast, estrogen signaling has a dramatic effect in enhancing cytokine-induced expansion of both the monocytic and granulocytic lineages of myeloid-derived suppressor cells (M-MDSCs and G-MDSCs). In addition, the immunosuppressive activity of specifically granulocytic MDSCs was significantly increased. The expansion of immature myeloid cells that acquire immunosuppressive activity in the periphery and at tumor beds is an universal occurrence in hosts with advanced solid tumors [3]. MDSCs are emerging as important contributors to immunosuppression, tumor angiogenesis and drug resistance [4]. We demonstrated that estrogen-induced accumulation of MDSCs was sufficient to accelerate tumor growth by blunting anti-tumor immunity. Accordingly, differences in the outcome of oophorectomized tumorbearing mice completely disappeared in mice lacking adaptive immunity.

To demonstrate the clinical relevance of estrogeninduced mobilization of MDSCs, we expanded immature myeloid cells from the bone marrow of different lung cancer patients in response to inflammatory cytokines typically upregulated in cancer patients. Again, blocking estrogen signaling with specific small molecule inhibitors had a significant effect on the mobilization of MDSCs with markers of both the monocytic and granulocytic lineages. These results have obvious implications for testing novel “complete” estrogen antagonists in cancer patients, and in particular in premenopausal women, regardless of the status of ER expression on the tumor cells. Although the role of immunosuppressive MDSCs in impairing the effectiveness of cancer immunotherapy remains poorly investigated, it is unlikely that tumorreactive T cells will be effective in the presence of a large myeloid inhibitory burden. While chronic inhibition of estrogen activity is not without side effects, cancer patients receiving immune checkpoint inhibitors could therefore benefit from short-term inhibition of estrogen-enhanced mobilization of immunosuppressive myeloid cells using complete antagonists, such as fulvestrant.

Mechanistically, we found that ERα activation is sufficient to enhance the STAT3 pathway in human and mouse bone marrow myeloid precursors. This occurs at least in part by increasing JAK2 and SRC activity. Because STAT3 activation is crucial for the expansion and increased survival of immature myeloid precursors [5], the use of novel JAK2 inhibitors offers great promise to prevent MDSC mobilization in the bone marrow, independently of their activities on tumor cells. Furthermore, we have recently reported that trametinib, an FDA-approved MEK inhibitor, effectively prevents the expansion specifically of M-MDSCs from human and mouse bone marrow precursors [6]. Although the expansion of G-MDSCs is severely increased upon estrogen signaling, but is unaffected by MEK inhibition, it will be of interest to determine whether the pSTAT3 activating effects of estradiol and the activation of the RAS pathway intersect, at in least M-MDSCs.

Besides downstream activation of JAK and SRC kinases, we did not find significant increases in IL-6 receptor expression on the cell surface upon estrogen signaling. However, it is theoretically possible that estrogen activity results in increased secretion of a soluble form of the IL-6R. Ongoing experiments are testing this possibility. In addition, while we found that ERα activation is sufficient to promote MDSC expansion, the role of ERβ signaling on hematopoietic (and even tumor) cells remains poorly understood. In some tumors, the activity of ERβ delays, rather than accelerates, tumor growth. It is therefore possible that ERβ agonists also offer therapeutic opportunities to block pathological myelopoiesis.

Our work also provides a mechanistic rationale for the seminal studies of Ostrand-Rosenberg and colleagues demonstrating the requirement of MDSCs for maternalfetal tolerance [7]. The authors demonstrate that during pregnancy, when the predominant form of estrogen changes to highly up-regulated estriol, MDSCs regulate many of the mechanisms previously attributed to maternalfetal tolerance and could therefore be used to assist pregnant women to avoid spontaneously miscarriages due to dysfunctional immunity at the maternal-fetal interface.

Finally, our results open new possibilities to better understand tumorigenesis in BRCA1/2-mutation carriers. In fact, BRCA1 haploinsufficiency results higher levels of estradiol [8], which could lead to increased accumulation of myeloid cells during inflammatory events associated with ovulation, or during tumor initiation.

Overall, our study demonstrates that estrogen signaling is a major driver of pathological myelopoiesis in cancer and probably in physiological conditions. Clinical testing should determine the synergy of anti-estrogens and emerging immunotherapies in cancer patients.
